# Homocysteine Impairs Endothelial Cell Barrier Function and Angiogenic Potential via the Progranulin/EphA2 Pathway

**DOI:** 10.3389/fphar.2020.614760

**Published:** 2021-01-08

**Authors:** Dan Tian, Qing Qin, Mingfei Li, Xiaoyu Li, Qing Xu, Qianzhou Lv

**Affiliations:** ^1^ Department of Pharmacy, Zhongshan Hospital, Fudan University, Shanghai, China; ^2^ Department of Cardiology, Zhongshan Hospital, Fudan University, Shanghai Institute of Cardiovascular Disease, Shanghai, China

**Keywords:** progranulin, EphA2, hyperhomocysteinemia, adhesion, VE-Cadherin

## Abstract

Hyperhomocysteinemia is a well-recognized independent risk factor for cardiovascular disease. To date, the mechanism of pathological plasma homocysteine (Hcy) level elevation remains to be elucidated. We aimed to investigate the levels of progranulin (PGRN), Eph-receptor tyrosine kinase-type A2 (EphA2), vascular cell adhesion molecule-1 (VCAM-1), and Hcy in patients with arteriosclerosis and investigate their functions in Hcy-injured human umbilical vein endothelial cells (HUVECs). EphA2 knockdown was induced in HUVECs by shRNA lentivirus infection with EphA2-RNAi, and bulk RNA-seq assay was performed. Then we investigated the mechanism underlying the effect of recombinant human PGRN (rhPGRN) combined with shRNA interference of EphA2 on cell proliferation, migration, and angiogenesis in Hcy-injured HUVECs. Results showed that serum EphA2, VCAM-1, and Hcy levels in acute coronary syndrome patients were significantly higher than those in chronic coronary syndrome patients (*p* = 0.000; *p* = 0.000; *p* = 0.033, respectively). *In vitro*, we demonstrated that knockdown of EphA2 significantly impaired cell adhesion and inhibited HUVECs migration and angiogenesis (*p* < 0.001), which was associated with reduction in VCAM1 and VE-cadherin (*p* < 0.05). Hcy modulated the expression of PGRN and EphA2 in a time-and dose-dependent manner. However, rhPGRN ameliorated the Hcy-induced reduction in cell viability and migration (*p* < 0.05). Mechanistically, we found that PGRN/EphA2 and its downstream AKT/NF-κB signaling might be the primary signal transduction pathways underlying Hcy-induced injury. The present study illustrated that PGRN plays a previously unrecognized role in Hcy-induced endothelial injury, which is achieved through its interaction with EphA2 signaling, implying a promising therapeutic target for cardiovascular disease.

## Highlights

1. Eph-receptor tyrosine kinase-type A2 (EphA2) knockdown alters adhesion molecules expression *in vitro*.
2. Homocysteine modulates the expression of progranulin and EphA2 in a time- and dose-dependent manner.
3. The progranulin/EphA2 axis might be the primary mechanism by which Hcy impairs endothelial adhesion and angiogenesis.

## Introduction

Hyperhomocysteinemia (HHcy) is considered a significant and independent risk factor for cardiovascular disease (CVD), stroke, and neurodegenerative disease (Clarke et al., 1991; Veeranna et al., 2011; Shi et al., 2015; Moretti and Caruso, 2019). Endothelial impairment, one of the earliest manifestations of cardiovascular damage, is associated with vascular injuries such as altered angiogenesis and increased permeability (Ross and Glomset, 1973; Park-Windhol and D’Amore, 2016). HHcy alters the endothelial cell (EC) barrier function and angiogenic potential; however, the molecular mechanisms remain elusive (Lai and Kan, 2015; Mohamed et al., 2017; Moretti and Caruso, 2019). Progranulin (PGRN) is a secreted glycoprotein encoded by GRN. PGRN is implicated in multiple pathological processes, such as regulation of inflammation, promotion of proliferation, regulation of cell cycle progression, cell motility, and regulation of neurotrophins and lysosomes (He and Bateman, 2003; Arrant et al., 2018). Kojima et al. first reported that PGRN is expressed in atherosclerotic plaques (Kojima et al., 2009). PGRN^(−/−)^ApoE^(−/−)^ mice exhibits more severe atherosclerotic lesions than PGRN^(+/+)^ApoE^(−/−)^ mice, possibly due to inflammatory cytokine and adhesion molecule accumulation as well as a reduction in endothelial nitric oxide synthase levels (Kawase et al., 2013). Interestingly, EphA2 was validated as a functional receptor of PGRN in an *in vitro* study, and EphA2 silencing significantly prevents PGRN-mediated autoregulation (Neill et al., 2016; Buraschi et al., 2020). Reduced progression to advanced atherosclerotic plaques in EphA2^(−/−)^ApoE^(−/−)^ mice suggests that EphA2 has an essential function in the early stage of cardiovascular disease (Neill et al., 2016; Cui et al., 2019). Fu et al. first revealed the therapeutic role of PGRN in HHcy-induced cardiorenal dysfunction (Fu et al., 2017). In addition, previous studies have demonstrated the potential effect of EphA2 and PGRN in altering vascular permeability and the inflammatory response in lung injury (Guo et al., 2012; Hong et al., 2015). However, whether PGRN and EphA2 are involved in vascular endothelial impairment induced by HHcy remains elucidated. This study aimed to investigate the levels of EphA2, PGRN, and Hcy in arteriosclerosis patients and to evaluate how PGRN and EphA2 exert their function in EC with Hcy-induced injury.

## Materials and Methods

### Patient Blood Samples

Blood samples were taken from patients with atherosclerosis admitted for coronary angiography in our center. All of the participants were enrolled in this study after signing the informed consent form. The protocol was approved by the Zhongshan Hospital Institutional Ethics Committee. Data were obtained for and compared between the acute coronary syndrome (ACS) group and chronic coronary syndrome (CCS) group. The expression of PGRN, EphA2, and vascular cell adhesion molecule-1 (VCAM1) was measured using enzyme-linked immunosorbent assay (ELISA) kits. Hcy levels were detected using the Homocysteine Assay Kit (circulating enzyme method, Nanjing Jiancheng Bioengineering Institute, China).

### Cell Culture and Regents

Human umbilical vein endothelial cells (HUVECs) were purchased from Sciencell Research Lab. HUVECs were cultured in endothelial cell medium (ECM) media containing 5% fetal bovine serum (FBS), 1% endothelial cell growth additive (ECGs), and 1% penicillin/streptomycin solution. THP-1 monocytes were cultured in RPMI-1640 medium (Sigma R8758) supplemented with 10% FBS. The cells were placed in an incubator at 37°C with a humidified atmosphere of 5% CO_2_. The cells were stimulated with DL-homocysteine (Sigma Aldrich) 2.0 mM in the presence or absence of recombinant human progranulin (rhPGRN) protein 200 ng/mL (R&D systems) for 24 h as well as treated with a variety of concentrations of rhPGRN (0, 50, 100, 200, 400 ng/mL) for 24 h depending on the experimental goals. The CCK8 kit and BCA protein assay kit were obtained from Beyotime (Beyotime Biotech, Shanghai, China). Granulin, VCAM1 antibodies were purchased from Abcam (Cambridge, United Kingdom), and VE-cadherin, p-EphA2 (Ser897), EphA2, p-AKT (Ser473), AKT, p-NF-κB p65 (Ser536), and NF-κB p65 antibodies were obtained from Cell Signaling Technology (Danvers, MA, United States).

### Quantitative Real-Time PCR

Quantitative real-time PCR was performed using total RNA extracted from different treatment groups using Trizol Reagent (T9424, Sigma-Aldrich). Then, cDNA was synthesized from 1,000 ng of total RNA using PrimeScript RT Master Mix (Takara Bio Inc., Kusatsu, Japan) and amplified using SYBR Premix Ex Taq II (Takara Bio Inc.). The PCR primers used are listed in Supplementary Table S1. GAPDH was used as a reference gene. Reactions were performed in triplicate under standard thermo cycling conditions using Quant Studio 5 (one cycle of 95°C for 30 s, followed by 40 cycles of 95°C for 5 s and 60°C for 34 s) with a melting curve, and the mean threshold cycle number was used.

### Generation of EphA2 Knockdown Cells

EphA2 knockdown was performed by transfecting cells with EphA2 (NM_004431) shRNA, which was inserted into the GV493 plasmid according to the instructions of GENEChem. The shRNA sequence for EphA2 was 5′-caG​GCT​GTG​TTG​AAG​TTC​ACT-3′. The sequence for the control shRNA (NC-RNAi) was 5′-TTC​TCC​GAA​CGT​GTC​ACG​T-3′. Transfection efficiency was determined by evaluating GFP expression under a microscope 48  and 72 h after transfection. Cells were selected with 1.5 μg/mL puromycin in ECM medium. Then, EphA2 expression levels were determined by immunoblotting using an appropriate antibody as previously described.

### Immunofluorescence Staining

Cells were seeded at a density of 5 × 10^5^cells/well in copolycoke dishes (NEST Biotechnology). After the cell fusion rate was greater than 50%, the cells were washed three times with cold phosphate-buffered saline (PBS), fixed with 4% paraformaldehyde and incubated in 0.5% Triton X-100 for 20 min. The cells were blocked in 1 mL Blocking Buffer for Immunol Staining (Beyotime Biotech, Shanghai, China) and then incubated with primary antibodies overnight. Three PBST (PBS containing Tween-20) washes were performed to remove unbound primary antibody, and then secondary antibodies conjugated to Alexa Fluor® 647 (1:500, Beyotime Biotech, Shanghai, China) were added. Finally DAPI staining solution (Beyotime Biotech, Shanghai, China) was used to counterstain the nuclei. Images were captured using an Olympus FV1000-D (BX) laser scanning confocal microscope (Olympus Life Science).

### Cell Adhesion

For the adhesion study, cells were plated into 6-well plates. Viable THP-1 monocytes were labeled with 1 μL Hoechst 33,342 (1,000×) (Beyotime Biotech, Shanghai, China) for 15 min and incubated with ECs for 30 min. The cells were fixed with 4% paraformaldehyde after rinsed three times with cold PBS. Six visual fields were selected under a fluorescent microscope, and cells were counted. Images were captured using an Olympus FV1000-D (BX) laser scanning confocal microscope (Olympus Life Science).

### Scratch Wound Assay

After transfection, HUVECs were plated at a density of 1 × 10^6^ cells/mL in a 6-well until the cell fusion rate reached 100%. A scratch was created with a P200 pipette tip, and the cells were washed three times with medium and then maintained in ECM containing 1% FBS in 5% CO_2_ at 37°C environment. Wounds were imaged at 24 and 48 h using a fluorescence microscope.

### Tubulogenesis Assay

HUVECs were collected with 0.25% trypsin/EDTA and seeded at a density of 4 × 10^5^ cells/well in 24 culture plates precoated with Matrigel matrix (Corning, NY, United States, 356234) and incubated at 37°C for 4∼6 h. Tubulogenesis was imaged under an inverted fluorescence microscope, and the results are expressed as the total tube length and mesh area relative to that of the control group by analyzing five representative fields. Independent experiments were performed in triplicate.

### Transwell Cell Migration Assay

For the Transwell cell migration assay, 500 µL medium containing 10% FBS was added to the lower chamber of Costar Transwell polycarbonate permeable supports (Corning, NY, United States). HUVECs (5 × 10^4^) in 200 µL medium containing 2% FBS and drugs were added to the upper chamber and incubated at 37°C for 6 h. The suspended cells were removed from the upper chamber, and the membranes were fixed in 4% paraformaldehyde. The migrated cells were stained with 0.1∼0.2% crystal violet and washed three times with PBS. The upper side of the chamber was gently wiped with a cotton swab and then dried at room temperature. Six representative fields were imaged using a fluorescence microscope. Independent experiments were performed in triplicate.

### Western Blotting

Cells were washed with cold PBS and lyzed in RIPA buffer (Beyotime Biotech, Shanghai, China) containing 1 mM phenylmethanesulfonyl fluoride (PMSF), protease inhibitors, and phosphatase inhibitor. Aliquots containing 20 µg of protein were separated by sodium dodecyl sulfate polyacrylamide gel electrophoresis and transferred onto PVDF membranes (Millipore). After blocking in 5% nonfat milk in Tris-buffered saline containing Tween-20 (TBST), the blots were incubated with primary antibodies overnight at 4°C followed by horseradish peroxidase-conjugated secondary goat anti-rabbit antibodies (1:6,000 dilution, Beyotime Biotech, Shanghai, China).

### Statistics

The data are expressed as the mean ± standard deviation (SD) or mean ± standard error of the mean (SEM). Statistical calculations were performed using GraphPad Prism 8 (GraphPad Software Inc.). Count data are presented as numbers and percentages. Demographic characteristics and medical histories, such as gender and disease, were analyzed by the Chi-squared test or Fisher’s exact test. Pearson linear correlation analysis or Spearman rank correlation coefficient analysis was used to analyze the correlation between two variables. Comparisons between two groups were analyzed by unpaired two-tailed Student’s t-test or the Mann-Whitney U test. Multiple groups were analyzed by one-way or two-way analysis of variance with Tukey’s post hoc test. A 2-sided *p* value < 0.05 was considered significant.

## Results

### Levels of EphA2, PGRN, VCAM1, and Hcy in ACS Patients

**FIGURE 1:**
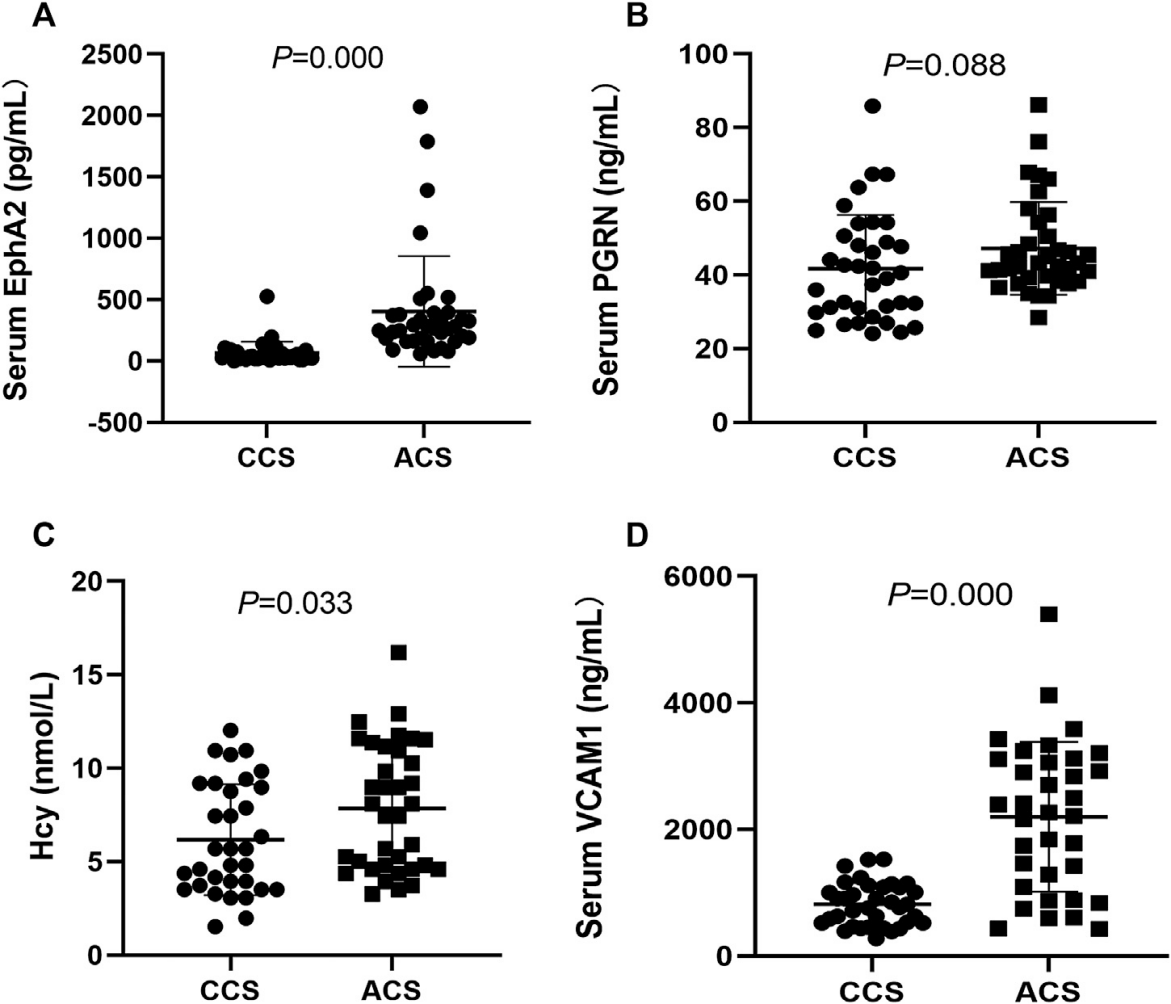
Comparison and correlation analysis of clinical indexes between the CCS patients (n = 36) and the ACS patients (n = 37). **(A–D)** Comparison of serum levels of EphA2, PGRN, VCAM1 and Hcy, respectively. CCS: chronic coronary syndrome; ACS: acute coronary syndrome. EphA2: Eph-receptor tyrosine kinase-type A2, PGRN: Progranulin, VCAM1: vascular cell adhesion molecule-1; Hcy: homocysteine.

The general data of the studied patients are shown in Supplementary Table S2. There was no significant difference in demographic characteristics or risk factors between the two groups (*p* > 0.05). The stenosis degree of the three-culprit lesion was higher in the ACS group than in the CCS group (*p* < 0.05). The levels of EphA2, VCAM1, and Hcy were significantly higher in the ACS group than in the CCS group (403.9 ± 449.4 vs. 67.4 ± 90.9, *p* = 0.000; 2,198.4 ± 1,182.9 vs. 816.2 ± 338.1, *p* = 0.000; and 7.8 ± 3.4 vs. 6.2 ± 3.0, *p* = 0.033, respectively). However, there was no significant difference in serum PGRN between the groups (47.2 ± 12.6 vs. 41.7 ± 14.6, *p* = 0.088) (Figures 1A–D).

### EphA2 Deficiency Suppressed Cell Adhesion Molecules Expressions

**FIGURE 2:**
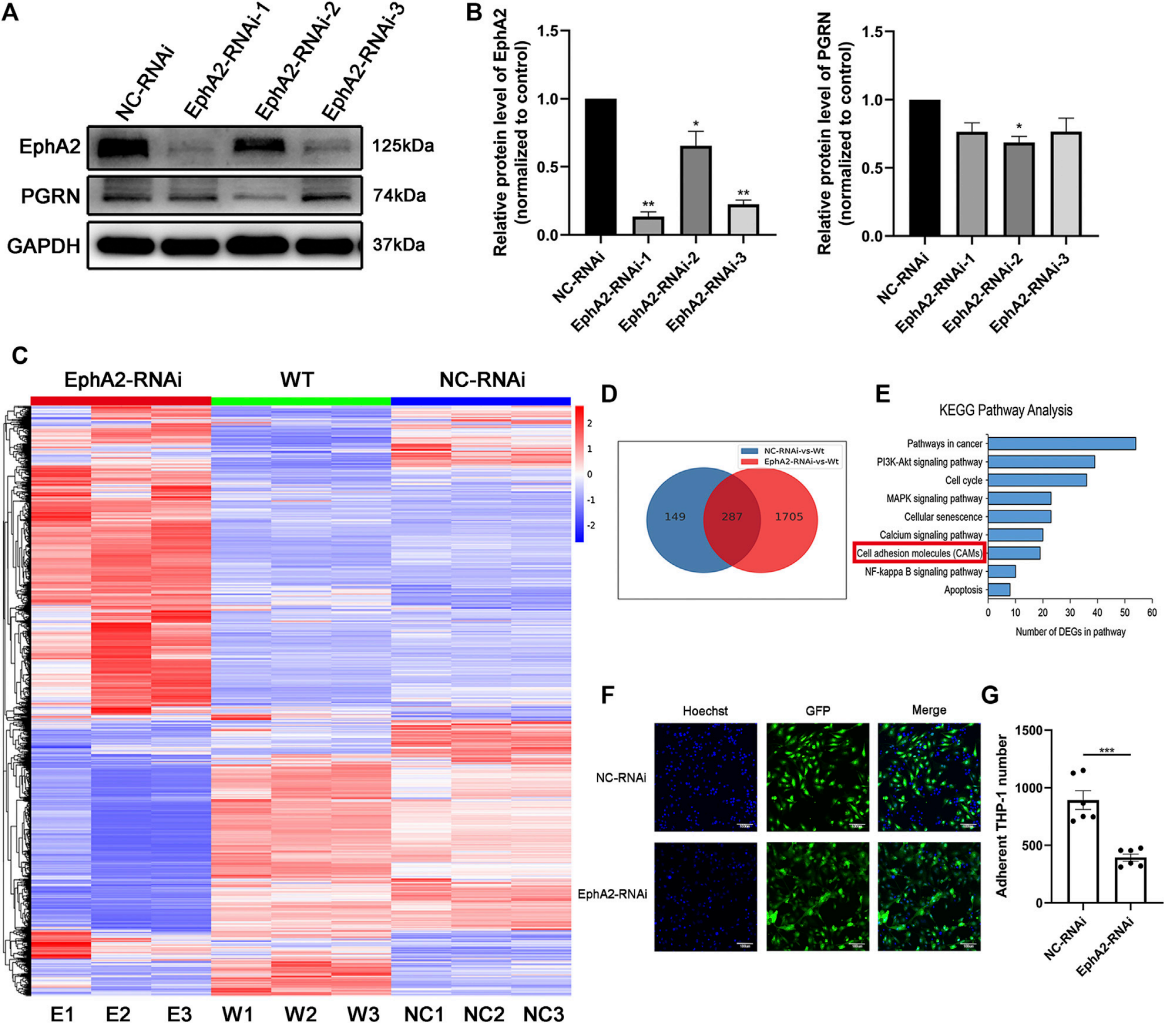
EphA2 knockdown suppressed cell adhesion molecule expression in HUVECs. **(A,B)** Western blotting results showed an efficient knockdown of EphA2 expression in HUVECs with different interference sequences (labeled as EphA2-RNAi-1, -2, -3) **(C)** Heat map of mRNA changes in EphA2-RNAi, NC-RNAi, and WT group by the bulk RNA-seq. **(D)** Venn diagrams depicted a total of 287 common differential genes in EphA2-RNAi cells vs. WT cells and NC-RNAi cells vs. WT cells **(E)** KEGG pathway assay of differential mRNA transcripts in EphA2-RNAi HUVECs identified by the RNA-seq. **(F)** THP-1 (blue, stained by Hoechst 33,342) monocyte adhesion in EphA2 knockdown HUVECs (GFP-labeled, green). **(G)** Comparison of adherent THP-1 number between EphA2-RNAi group and NC-RNAi group in HUVECs. Data are presented as means ± SEM (*p < 0.05, **p < 0.01, ***p < 0.001 vs. NC-RNAi group, ANOVA, n = 3 or 6). Scale bar: 100 μm. WT: wild-type. NC: negative control.

To investigate the potential function of EphA2, we further studied the endothelial reaction of HUVECs in response to transfection with three different EphA2-shRNA inserted into the GV493 plasmid (Figures 2A,B; Supplementary Figure S1). We took nine HUVECs samples to conduct bulk RNA-seq (EphA2-RNAi cells, NC-RNAi cells, and wild-type cells, each analyzed in triplicate). A total of 287 common differentially expressed genes were identified in EphA2-RNAi and NC-RNAi cells vs. wild-type cells. Kyoto Encyclopedia of Genes and Genomes (KEGG) pathway analysis of the differentially expressed mRNAs showed that the expression of cell adhesion molecules changed in the EphA2-RNAi group compared to the NC-RNAi group (Figures 2C–E). Consistent with the RNA-seq results, depletion of endothelial EphA2 altered EC migration and attenuated THP-1 monocyte adhesion *in vitro* (*p* < 0.001) (Figures 2F,G).

### EphA2 Depletion Suppressed HUVECs Migration and Angiogenesis

**FIGURE 3:**
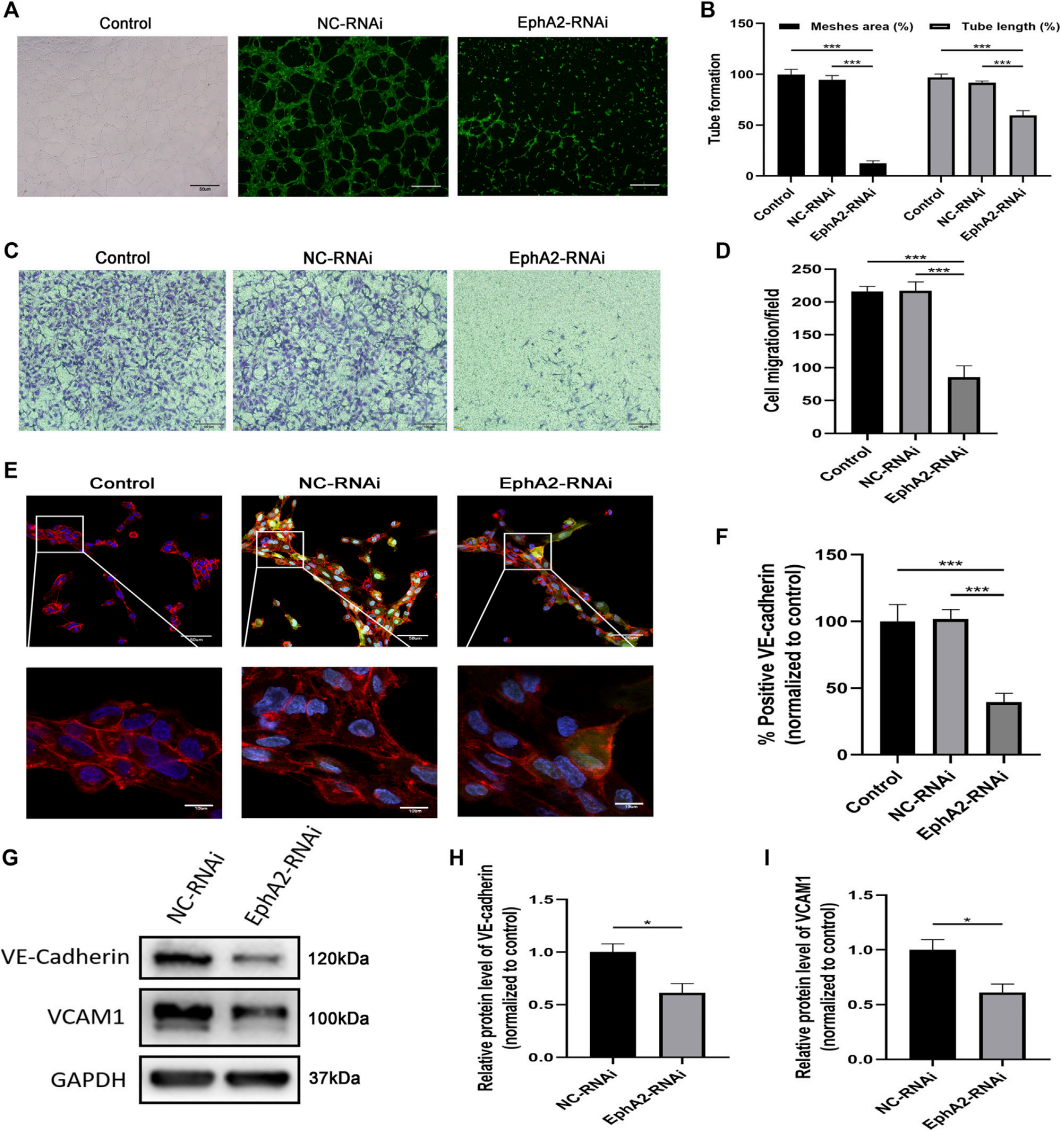
EphA2 depletion suppressed HUVECs migration and angiogenesis. **(A,B)** Results of tube formation assay of EphA2 knockdown cells by lentivirus infection compared with NC-RNAi cells and wild-type control. Scale bar, 50 μm **(C,D)** The cell migration was evaluated by the Transwell cell migration assay between groups mentioned above. Scale bar, 100 μm. **(E)** The distribution of VE-cadherin (red) in the cell membrane and DAPI (blue) was observed by immunofluorescence confocal microscopy in EphA2 knockdown HUVECs (GFP-labeled, green). Scale bar, 50 and 10 μm. **(F)** Statistical results of fluorescence intensity of VE-cadherin (normalized to control) in HUVECs. Fluorescence intensity was calculated with six randomly selected cells from the visual field. **(G–I)** The expression of VE-cadherin and VCAM1 were detected by Western blotting assay in EphA2 knockdown cells and NC-RNAi cells. Data are presented as means ± SEM (*p < 0.05, **p < 0.01, ***p < 0.001 vs. vehicle control, ANOVA, n = 3 or 6).

In addition, we observed inhibition of migration and angiogenesis abilities after transfecting HUVECs with EphA2-siRNA (*p* < 0.001 vs. vehicle control; *p* < 0.001 vs. NC-RNAi groups) (Figures 3A–D). VE-cadherin is a unique molecule expressed explicitly on the surface of vascular ECs. In this study, immunofluorescence analysis of VE-cadherin showed that the volume of HUVECs was significantly decreased in EphA2 knockdown cells. Cell morphology was changed considerably, as dissociated EC adhesion junctions and reduced chain-like staining of VE-cadherin in the cell membrane were observed in the EphA2 knockdown cells, whereas chain-like staining and tight junctions were seen in the vehicle control and NC-RNAi groups (Figures 3E–F). VCAM1 and VE-cadherin staining were significantly decreased in the EphA2 knockdown cells compared with NC-RNAi cells (*p* < 0.05) (Figures 3G–I).

### Decreased Levels of PGRN, EphA2, and VCAM1 After DL-Hcy Treatment for 24 h

**FIGURE 4:**
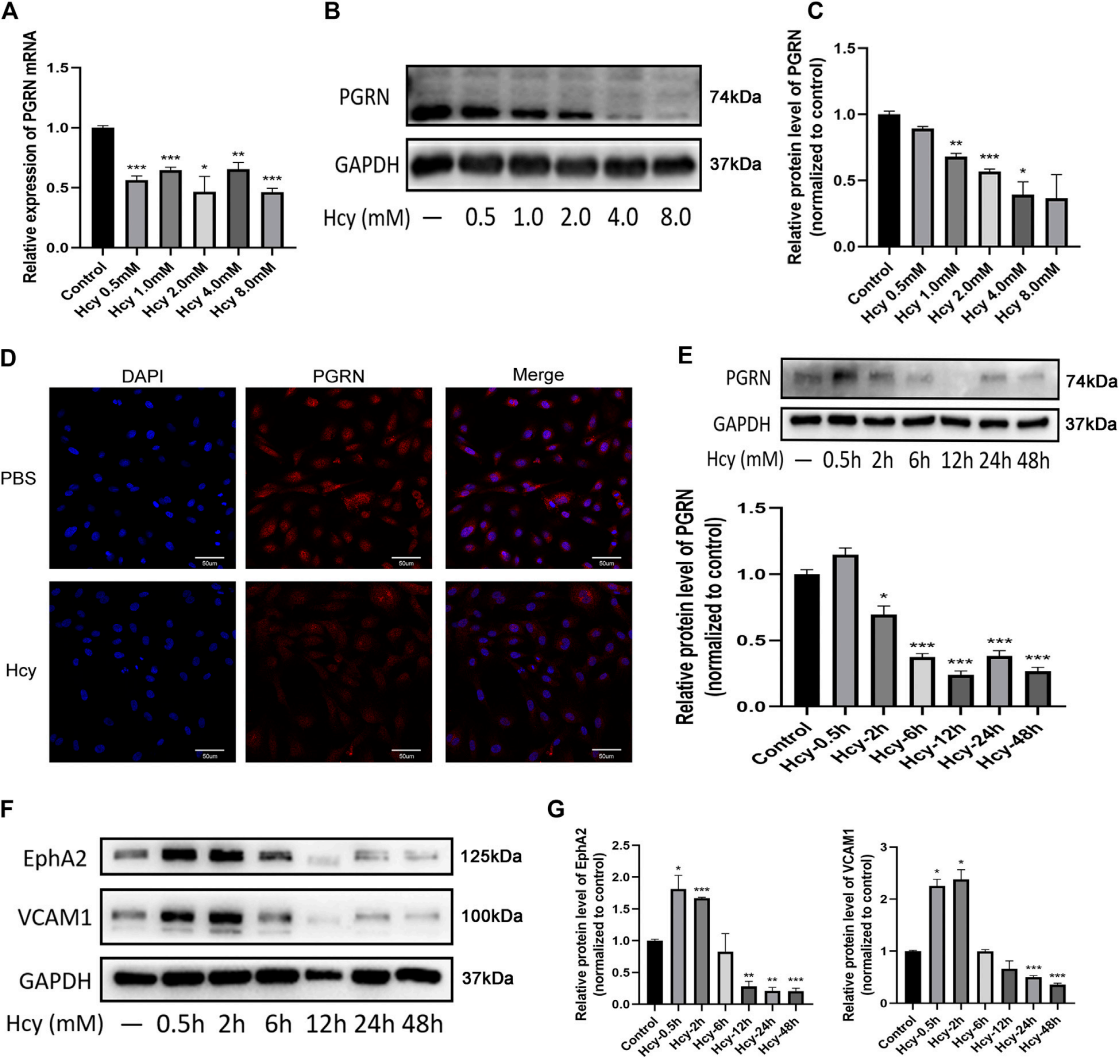
Decreased levels of PGRN, EphA2, and VCAM1 after DL-Hcy treatment. **(A)** The Real-time PCR analysis of PGRN in HUVECs treated with different concentrations of Hcy **(B,C)** Western blot analysis of PGRN in the absence and presence of Hcy (0.5, 1.0, 2.0, 4.0, and 8.0 mM) for 24 h. **(D)** Immunofluorescence result of the distribution of PGRN (red) and DAPI (blue) in the absence and presence of Hcy 2.0 mM for 24 h. Scale bar, 50 μm. **(E)** Western blotting analysis of PGRN stimulated with 2.0 mM Hcy at different time points (0.5, 2, 6, 12, 24, and 48 h). **(F,G)** Western blotting analysis of EphA2 and VCAM1 levels stimulated with 2.0 mM Hcy at different time points (0.5, 2, 6, 12, 24, and 48 h). Data are presented as means ± SEM (*p < 0.05, **p < 0.01, ***p < 0.001 vs. vehicle control, ANOVA, n = 3).

The results of bulk RNA-seq analysis of Hcy-injured HUVECs showed that Hcy influenced the pathway related to adherent junctions and focal adhesions (Supplementary Figure S2). We next evaluated the influence of Hcy on the expression of PGRN, EphA2, and VCAM1 in HUVECs (Figure 4). Western blotting analysis and immunofluorescence results showed that Hcy statistically down-regulated the expression of PGRN in a time- and concentration-dependent manner (*p* < 0.05) (Figures 4B–E). The expression of EphA2 and VCAM1 in HUVECs was up-regulated within 2 h after Hcy treatment and were down-regulated after 2 h following Hcy treatment (*p* < 0.05). Additionally, the expression of EphA2 and VCAM1 was altered simultaneously over time after Hcy treatment (Figures 4F,G).

### RhPGRN Ameliorated the Hcy-Induced Reduction in Cell Viability and Migration

**FIGURE 5:**
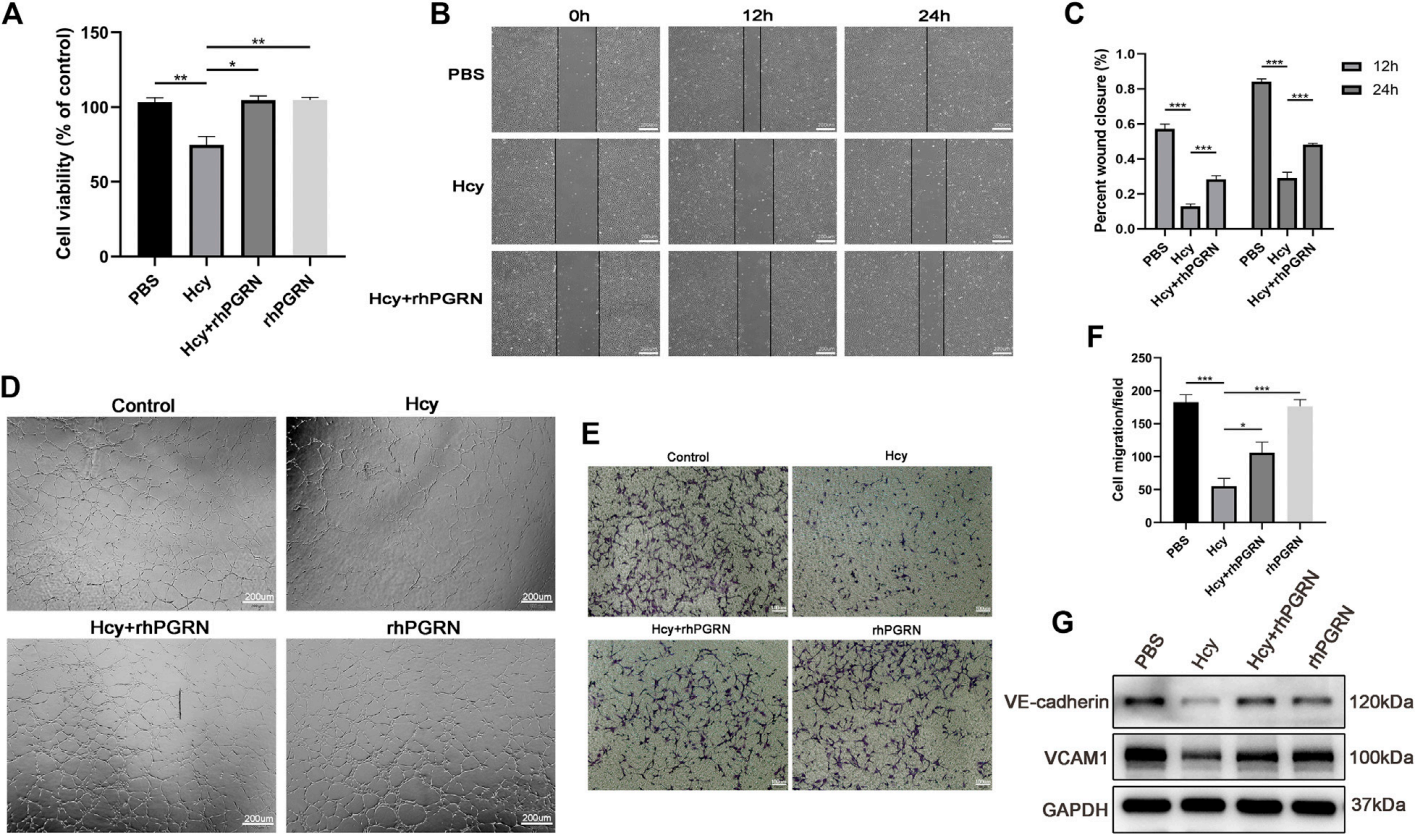
RhPGRN ameliorated the Hcy-induced reduction in cell viability and migration. **(A)** Results of cell viability in HUVECs after stimulated with 2.0 mM Hcy in the presence or absence of 200 ng/mL rhPGRN **(B,C)** Results of the scratch wound assay were recorded and calculated at 12 and 24 h after stimulated with 2.0 mM Hcy in the presence or absence of 200 ng/mL rhPGRN. Scale bar, 200 μm. **(D)** The tube formation assay of HUVECs stimulated with 2.0 mM Hcy, 200 ng/mL rhPGRN, and Hcy + rhPGRN for 6 h. Scale bar, 200 μm **(E,F)** The cell migration was evaluated by the Transwell cell migration assay in HUVECs stimulated with 2.0 mM Hcy, 200 ng/mL rhPGRN, and Hcy + rhPGRN for 6∼8 h. Scale bar, 100 μm. **(G)** Western blotting analysis of the protein levels of VCAM1 and VE-cadherin stimulated with 2.0 mM Hcy in the presence or absence of 200 ng/mL rhPGRN for 24 h. Data are presented as means ± SEM (*p < 0.05, **p < 0.01, ***p < 0.001 vs. vehicle control, ANOVA, n = 3 or 6).

Based on the above experiments, we next assessed whether rhPGRN exerts a protective effect on Hcy-induced EC injury. Hcy caused a significant reduction in cell viability, which was ameliorated in the Hcy plus rhPGRN group (*p* < 0.01) (Figure 5A). There were no statistically difference in HUVECs migration and angiogenesis between cells administrated with exogenous rhPGRN alone and corresponding cells administrated with PBS (*p* < 0.05). The results of the scratch wound assay, tube formation assay, and the Transwell cell migration assay further confirmed the capacity of rhPGRN to modulate EC adhesion (Figures 5B–F). Then, we verified the influence of rhPGRN by measuring the protein levels of VCAM1 and VE-cadherin under the same conditions. VCAM-1 and VE-cadherin were barely detectable in the group treated with Hcy for 24 h compared with the PBS group; however, rhPGRN restored the expression levels of VCAM-1 and VE-cadherin (Figures 5G). The above findings showed that PGRN might be one of the critical cytokines for Hcy-induced adhesion injury.

### The PGRN/EphA2 Axis Participated in Hcy-Induced Impairment of Endothelial Adhesion

**FIGURE 6:**
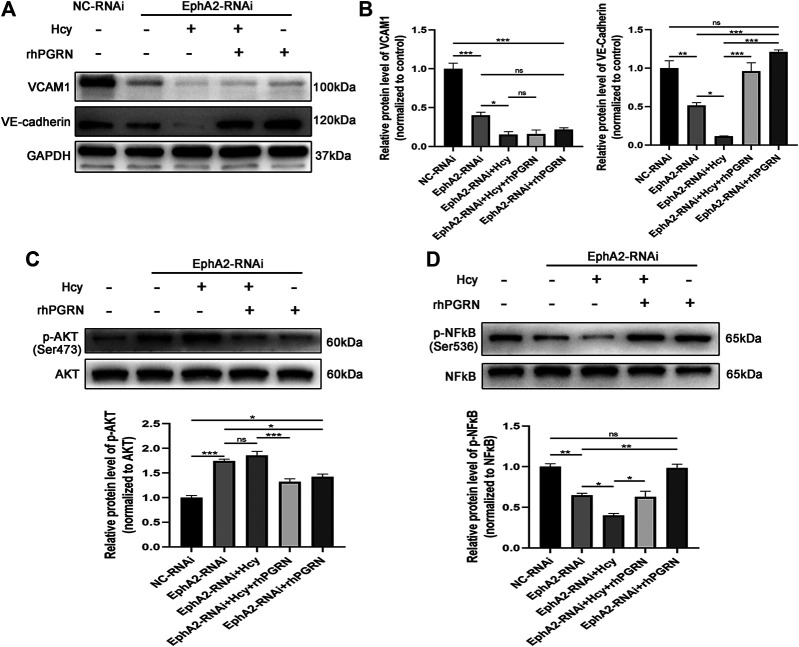
Effects of rhPGRN combined with shRNA interference of EphA2 on adhesion molecules and cytokines and NF-κB p65 and AKT pathways in Hcy-injured HUVECs. **(A)** Cells interfered with EphA2-shRNA (EphA2-RNAi) or NC-shRNA (NC-RNAi), and then the EphA2-RNAi group was treated with Hcy 2.0 mM, 200 ng/mL rhPGRN, Hcy + rhPGRN for 24 h. Representative Western blotting results of protein levels of VE-cadherin and VCAM1 between groups. **(B)** The summarized data of the protein levels of VCAM1 and VE-cadherin **(C)** Representative Western blotting gel documents and summarized data showing the levels of phospho-AKT (Ser473) and total AKT. **(D)** Representative western blot gel documents and summarized data showing the levels of p-NF-κB p65 (Ser536) and total NF-κB p65. Data are presented as means ± SEM (*p < 0.05, **p < 0.01, ***p < 0.001 vs. NC-RNAi group, ANOVA, n = 3). NC: negative control.

We next investigated the mechanism underlying the effect of rhPGRN combined with shRNA interference of its receptor EphA2 on adhesion molecules and cytokines and the NF-κB p65 and AKT pathways in Hcy-injured HUVECs. In line with previous results, knockdown of EphA2, as well as Hcy treatment, aggravated the loss of VCAM1 and VE-cadherin; however, administration of 200 ng/mL rhPGRN elevated the expression of VE-cadherin in EphA2 knockdown HUVECs. However, VCAM1 levels were barely increased after rhPGRN treatment in EphA2-RNAi HUVECs in both the presence and absence of Hcy (Figures 6A,B). Subsequently, we found distinct changes in the expression of AKT and NF-κB p65 and the phosphorylation of these proteins between the NC-RNAi group and the EphA2-RNAi group (Figures 6C,D). Additionally, Hcy treatment aggravated the changes in the phosphorylation levels of AKT and NF-κB p65, which were restored to normal levels by exogenous rhPGRN.

### Autoregulation Between PGRN and EphA2 was Activated by Exogenous rhPGRN

**FIGURE 7:**
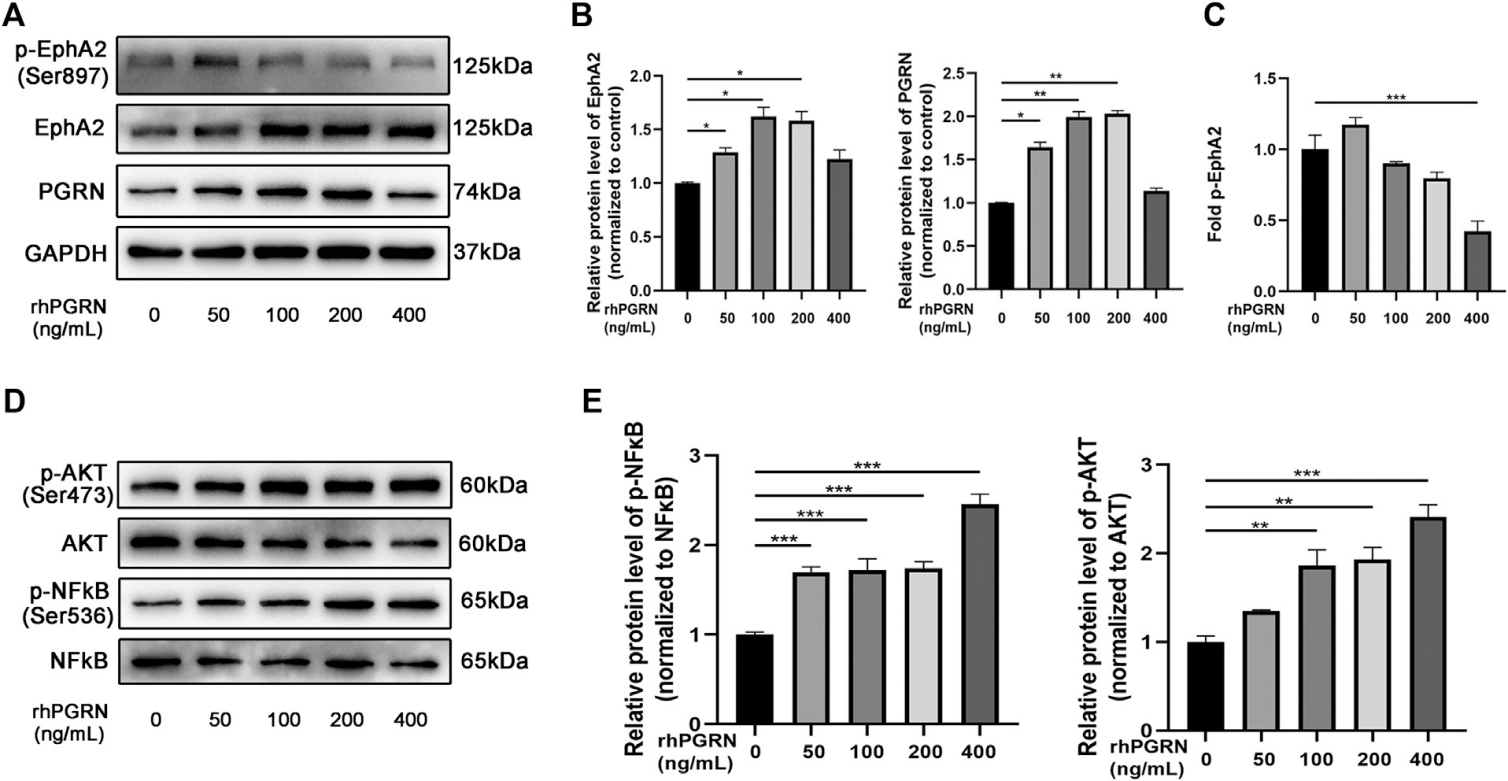
The effect of exogenous rhPGRN on the PGRN/EphA2 axis and the NF-κB p65 and AKT pathways. **(A)** HUVECs were administered with a variety of concentrations of rhPGRN (0, 50, 100, 200, 400 ng/mL) for 24 h. Western blotting gel documents of p-EphA2 (Ser897), EphA2, and PGRN showed relative protein levels in different groups. **(B)** The summarized data of the protein levels of EphA2 and PGRN **(C)** The summarized data of the protein levels of fold p-EphA2 (Ser897). **(D)** Phospho-AKT (Ser473), total AKT, NF-κB p65 (Ser536), and total NF-κB p65 signaling molecules were detected by the Western blotting. **(E)** The summarized data the levels phospho-NF-κB p65 (Ser536), total NF-κB p65. Data are presented as means ± SEM (*p < 0.05, **p < 0.01, ***p < 0.001 vs. vehicle, ANOVA, n = 3).

To further clarify the mechanism of the interaction between PGRN and EphA2, we applied rhPGRN at a variety of concentrations (0, 50, 100, 200, and 400 ng/mL) for 24 h to assess the effects of these concentrations *in vivo*. We found that the administration of rhPGRN at 200 ng/mL or less increased the expression of PGRN and EphA2 in a dose-dependent manner (*p* < 0.05) (Figures 7A,B). However, p-EphA2 (Ser897)/EphA2 expression was increased by a low dose of rhPGRN (*p* > 0.05) and significantly decreased by 400 ng/mL rhPGRN (*p* < 0.001), as shown in Figure 7C. The levels of the signaling molecules NF-κB p65 and AKT were measured by Western blotting. Phosphorylation of NF-κB p65 and AKT was significantly increased by a high dose of rhPGRN (*p* < 0.01) (Figures 7D,E).

## Discussion

For the first time, we investigated the levels of EphA2, PGRN, VCAM1, and Hcy in arteriosclerosis patients and evaluated the function of PGRN and EphA2 in Hcy-induced injury of ECs. There are four essential findings of this study: 1) EphA2 knockdown impairs vascular growth and migration and changes adhesion molecule expression *in vitro*. 2) Hcy modulates the expression of PGRN and EphA2 in a time-and dose-dependent manner. 3) RhPGRN ameliorates the Hcy-induced reduction in cell viability and migration, and might increase endothelial barrier function recovery by regulating the expression of VCAM1 and VE-cadherin. 4) Mechanistically, the PGRN/EphA2 axis might be the primary mechanism by which Hcy impairs endothelial adhesion and angiogenesis.

Adhesion and inflammatory molecules, such as VCAM-1 and intercellular adhesion molecules-1 (ICAM-1), are crucial players in the progression of atherosclerosis. Several biomechanical stimuli predominantly induce endothelium activation via pleiotropic transcription factor NF-κB-dependent ICAM-1 and VCAM-1 expression on the cell surface, resulting in leukocyte recruitment and extravasation (Desideri and Ferri, 2005; Jaipersad et al., 2014). It was previously reported that EphA2 knockdown inhibits NF-κB activation and impairs endothelial permeability (Carpenter et al., 2012). In addition, multiple lines of evidence suggest that PGRN deficiency weakens the adhesion of and tight junctions between endothelial cells and tumor cells, leading to barrier disruption or metastasis (Jackman et al., 2013; Zhao et al., 2020). Taken together, we believe that EphA2 might be a crucial player in the initiation of the atherosclerosis process, specifically the recruitment of leukocytes to sites of inflammation. In line with previous studies, the levels of the soluble isoform of EphA2 and VCAM1 in the plasma were significantly higher in the ACS group than in the CCS group, but the difference in PGRN levels between groups did not reach significance (De Caterina et al., 1997; Nakashima et al., 1998; Radecke et al., 2015).

VE-cadherin is an adhesion molecule that is exclusively expressed in the endothelium, forms adhesive contacts and regulates endothelial permeability (Vincent et al., 2004). Additionally, VE-cadherin and EphA2 are co-localized in cell-cell adhesion junctions in aggressive melanoma (Hong et al., 2015). Our data showed that depletion of endothelial EphA2 decreased EC migration, decreased THP-1 monocyte adhesion, and impaired angiogenesis *in vitro*. In EphA2-RNAi cells, cell morphology was changed considerably, as discontinuous endothelial junctions and reduced chain-like staining of VE-cadherin in the cell membrane, which ultimately increased the permeability of ECs, were observed. Collectively, our data show that the angiogenic potential and migration-promoting capacity of EphA2 are associated with VE-cadherin and VCAM1.

HHcy is an independent risk factor for vascular disease; however, the mechanism is not clear. Recent studies have revealed that VE-cadherin is involved in the Hcy-induced increase in vascular permeability increase (Muradashvili et al., 2014). In addition, Hcy can sensitize HUVECs to the effect of inflammatory mediators (thrombin and LPS), partly through VCAM-1 expression (Seguin et al., 2008). The vascular endothelium plays a prominent role in regulating membrane permeability, maintaining vascular homeostasis and modulating many physiological processes, such as angiogenesis (Verrier and Boyle, 1996). As expected, we found that Hcy treatment caused a decrease in PGRN levels in a time- and dose-dependent manner. The expression of EphA2 and VCAM1 also decreased after Hcy treatment. Angiogenesis destabilization and migration impairment were consistent with the reduction in membrane-bound VE-cadherin and VCAM1 in the Hcy group. However, rhPGRN ameliorates the Hcy induced reduction in cell viability and adhesion. Overall, PGRN has a specialized function in EC adhesion, as it is involved in the regulation of VCAM1 and VE-cadherin in endothelial cells.

RhPGRN treatment in the presence or absence of Hcy resulted in faster recovery of VE-cadherin levels in EphA2-RNAi cells than in NC-RNAi cells, whereas VCAM1 resulted in a low recovery rate. These results suggested that the protective effects of PGRN on cell adhesion and angiogenesis might depend on the normal expression of EphA2. Neill et al. also found that PGRN could bind EphA2 and activate MAPK and AKT signaling pathways through the binding (Neill et al., 2016). In line with a previous study, the phosphorylation of NF-κB was decreased in the EphA2-RNAi group compared to the NC-RNAi group (Carpenter et al., 2012). However, we found that the phosphorylation of AKT was elevated after EphA2 knockdown. In addition, Hcy treatment aggravated the changes in the phosphorylation levels of AKT and NF-κB, which were restored to normal levels by exogenous rhPGRN. In addition, we noticed that exogenous rhPGRN could increase the expression of VE-cadherin in EphA2 knockdown cells, which might be explained by the hypothesis that PGRN was responsible for the maintenance of VE-cadherin, thereby regulating the junctional adhesive strength of ECs.

Previous studies have found that PGRN could be decomposed into small peptides (GRNs), which plays the opposite role compared with PGRN in inflammation regulation (Zhu et al., 2002; Paushter et al., 2018). Thus, we administered rhPGRN at a variety of concentrations to HUVECs to simulate the effect of different plasma concentrations of PGRN on vascular endothelial cells *in vivo*. In this study, rhPGRN induced the expression of PGRN and EphA2 in a dose-dependent manner. PGRN-mediated autoregulation might be associated with the expression of p-EphA2 (Ser897)/EphA2. P-EphA2 (Ser897)/EphA2 was changed in different manners by different doses of rhPGRN (increased phosphorylation of EphA2 at a low dose and decreased phosphorylation at a high dose). We found that phosphorylation of NFκB p65 and AKT was significantly increased in a dose-dependent manner.

**FIGURE 8:**
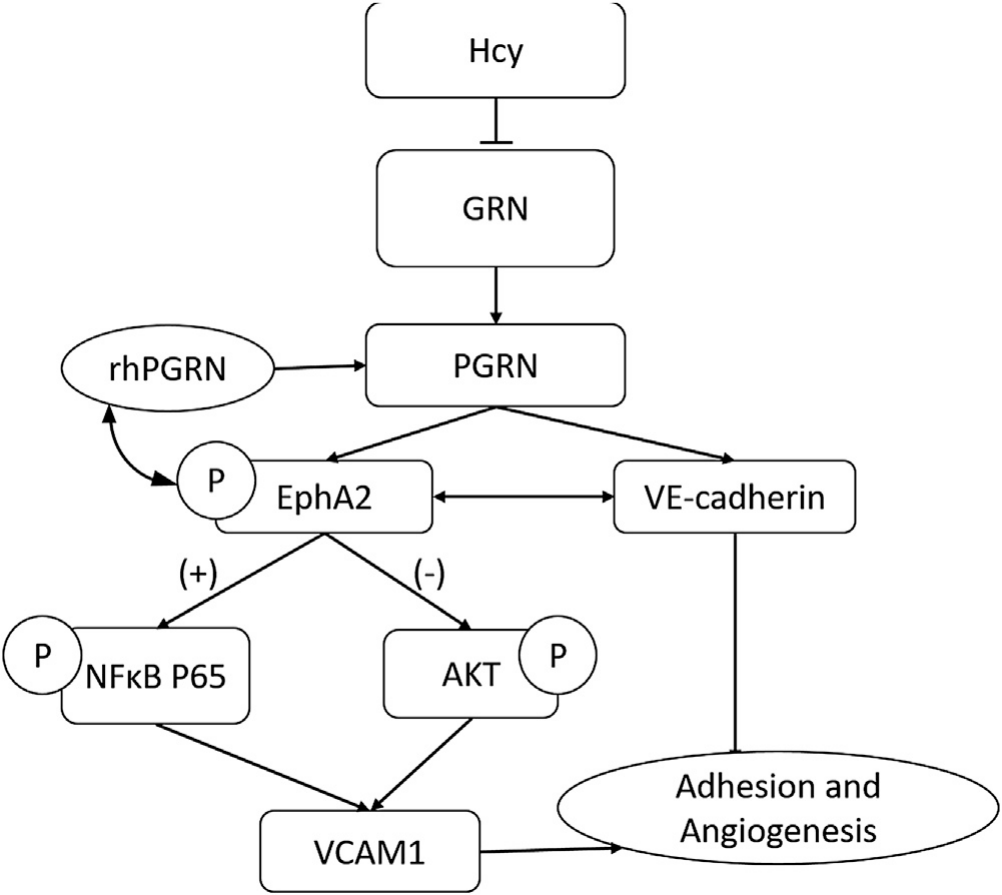
The PGRN/EphA2 axis might be one of the primary mechanisms by which Hcy impairs endothelial adhesion and angiogenesis.

Mechanistically, the PGRN/EphA2 axis might be one of the primary mechanisms by which Hcy impairs endothelial adhesion and angiogenesis (Figure 8). The regulatory pattern of PGRN expression in the content of diseases remains complex and context-specific. PGRN and EphrinA1 might coexist as ligands for EphA2 and maintain a relative balance under physiological conditions until inflammation disturbs this balance. More detailed study designs are needed to explore the specific molecular mechanism of the PGRN/EphA2 axis in endothelial homeostasis.

## Conclusion

In summary, our findings illustrate that PGRN exerts a previously unrecognized role in Hcy-induced endothelial injury, which is achieved through the interaction of EphA2 signaling, implying that it is a prognostic marker or a promising therapeutic target for cardiovascular disease.

## Data Availability

The raw data supporting the conclusions of this article will be made available by the authors, without undue reservation, to any qualified researcher.
