# Supplementary material for: Homocysteine Impairs Endothelial Cell Barrier Function and Angiogenic Potential via the Progranulin/EphA2 Pathway
Source: Front Pharmacol. 2021 Jan 8;11:614760. doi: 10.3389/fphar.2020.614760 (PMC7836014; doi:10.3389/fphar.2020.614760)
Supplement: Supplementary file 1 [file table1.docx]

Table 1 The PCR primers list

| Primer name | 5’ to 3’ |
| --- | --- |
| GAPDH forward | GAAGGTGAAGGTCGGAGTC |
| GAPDH reverse | GAAGATGGTGATGGGATTTC |
| PGRN forward | ATCTTTACCGTCTCAGGGACTT |
| PGRN reverse | CCATCGACCATAACACAGCAC |
| EphA2 forward | ATGACCAACGACGACATCAA |
| EphA2 reverse | GCAGGGGGAGGAAAGAACTA |

Table 2 Baseline characteristics and coronary angiography results of patients

| Variable | ACS group (n=37) | CCS group (n=36) | *p* value | *OR (95%CI)* |
| --- | --- | --- | --- | --- |
| Males, n (%) | 31 (83.8) | 23 (63.8) | 0.065 | 2.920（1.029-9.246） |
| Age, y (mean ± SD) | 65.8 ± 11.6 | 62.8 ± 10.4 |  |  |
| BMI (kg/m^2^) | 25.1 ± 3.2 | 24.6 ± 3.6 |  |  |
| Cardiovascular risk factor | | | | |
| Hypertension, n (%) | 22 (59.5) | 21 (58.3) | 1.000 | 1.048 (0.4205-2.616) |
| Diabetes mellitus, n (%) | 14 (37.8) | 8 (22.2) | 0.203 | 2.130 (0.7421-6.311) |
| Smoking, n (%) | 19 (51.4) | 11 (30.6) | 0.097 | 2.399 (0.9551-5.881) |
| Drinking, n (%) | 5 (13.5) | 5 (13.9) | 1.000 | 0.9688 (0.2741-3.426) |
| Culprit lesion | | | | |
| LAD (%) | 69.0 ± 32.3 | 22.5 ± 19.6 | 0.000 |  |
| Cx (%) | 37.8 ± 38.7 | 8.3 ± 15.9 | 0.000 |  |
| RCA (%) | 65.6 ± 39.8 | 8.9 ± 16.3 | 0.000 |  |

Data presented as mean ± SD or n (%). SD: standard deviation; ACS: acute coronary syndrome; CCS: chronic coronary syndrome; OR: odds ratio; CI: confidence interval; BMI: body mass index; LAD: left anterior descending artery; CX: circumflex artery; RCA: right coronary artery.


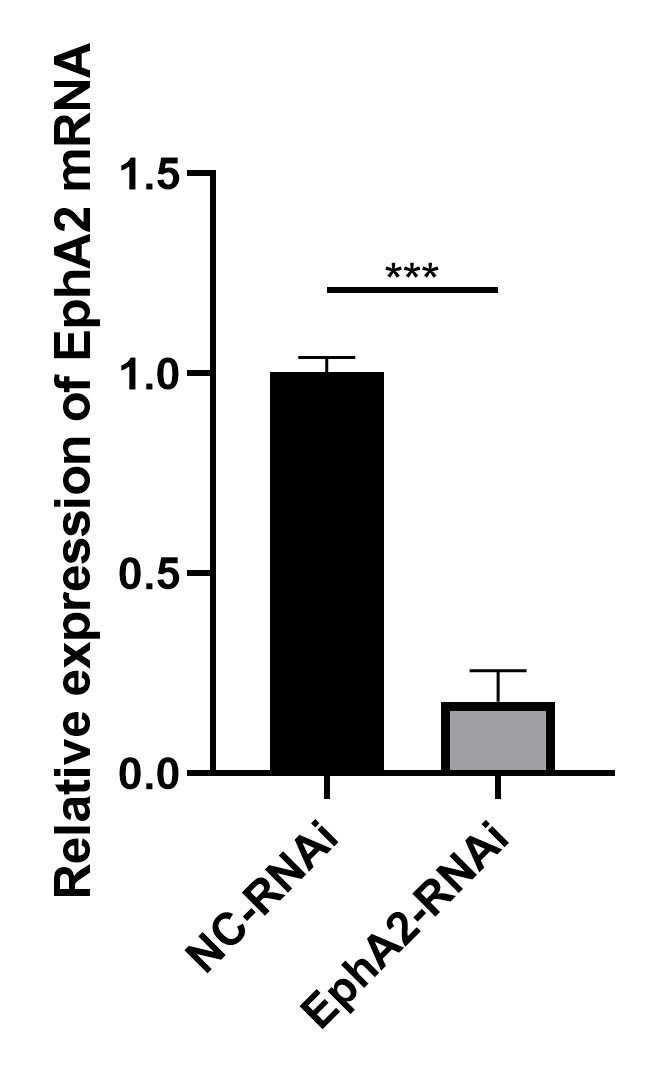


Figure 1 The PCR results of mRNA level changes of EphA2 in the EphA2-RNAi HUVECs (transfecting cells with EphA2 shRNA) compared with that of the NC-RNAi HUVECs (transfecting cells with negative control shRNA). Data are presented as means ± SEM. The RT-qPCR analyses were performed 3 times (***p < 0.001 vs. NC-RNAi).


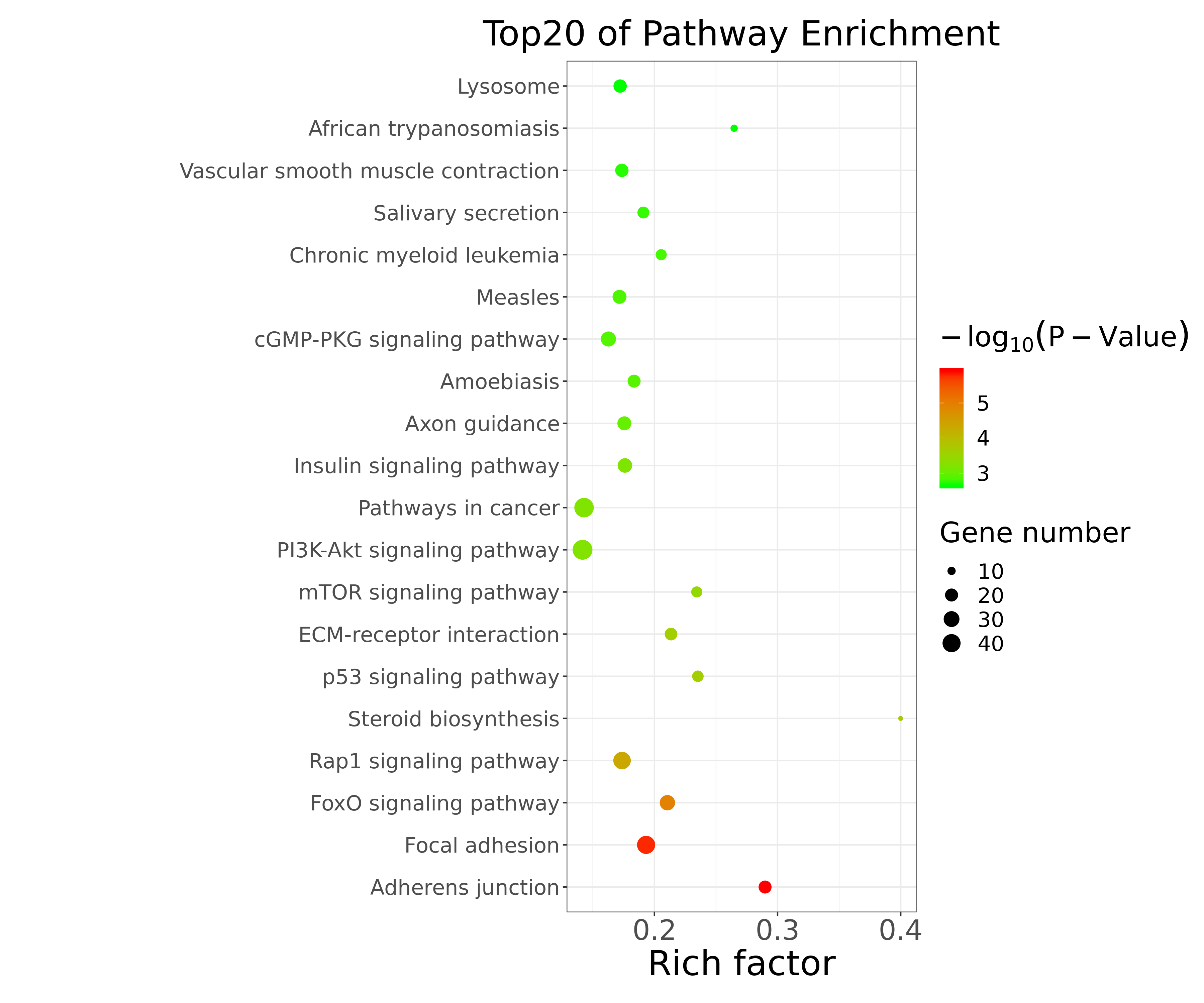


Figure 2 Bulk RNA-seq results showed the Top20 pathway enrichment of the Homocysteine-injured HUVECs compared to the control group. Homocysteine significantly decreased adherens junction and focal adhesion.
